# Utility of neuron-specific enolase in traumatic brain injury; relations to S100B levels, outcome, and extracranial injury severity

**DOI:** 10.1186/s13054-016-1450-y

**Published:** 2016-09-08

**Authors:** Eric Peter Thelin, Emma Jeppsson, Arvid Frostell, Mikael Svensson, Stefania Mondello, Bo-Michael Bellander, David W. Nelson

**Affiliations:** 1 Department of Clinical Neuroscience, Karolinska Institutet, Stockholm, Sweden; 2 Department of Neurosurgery, Karolinska University Hospital, Stockholm, Sweden; 3 Department of Biomedical and Dental Sciences and Morphofunctional Imaging, University of Messina, Messina, Italy; 4 Department of Physiology and Pharmacology, Section of Anesthesiology and Intensive Care, Karolinska Institutet, Stockholm, Sweden

**Keywords:** Traumatic brain injury, Biomarkers, S100B, Neuron-specific enolase, Outcome

## Abstract

**Background:**

In order to improve assessment and outcome prediction in patients suffering from traumatic brain injury (TBI), cerebral protein levels in serum have been suggested as biomarkers of injury. However, despite much investigation, biomarkers have yet to reach broad clinical utility in TBI. This study is a 9-year follow-up and clinical experience of the two most studied proteins, neuron-specific enolase (NSE) and S100B, in a neuro-intensive care TBI population. Our aims were to investigate to what extent NSE and S100B, independently and in combination, could predict outcome, assess injury severity, and to investigate if the biomarker levels were influenced by extracranial factors.

**Methods:**

All patients treated at the neuro-intensive care unit at Karolinska University Hospital, Stockholm, Sweden between 2005 and 2013 with at least three measurements of serum S100B and NSE (sampled twice daily) were retrospectively included. In total, 417 patients fulfilled the criteria. Parameters were extracted from the computerized hospital charts. Glasgow Outcome Score (GOS) was used to assess long-term functional outcome. Univariate, and multivariate, regression models toward outcome and what explained the high levels of the biomarkers were performed. Nagelkerke’s pseudo-R^2^ was used to illustrate the explained variance of the different models. A sliding window assessed biomarker correlation to outcome and multitrauma over time.

**Results:**

S100B was found a better predictor of outcome as compared to NSE (area under the curve (AUC) samples, the first 48 hours had Nagelkerke’s pseudo-R^2^ values of 0.132 and 0.038, respectively), where the information content of S100B peaks at approximately 1 day after trauma. In contrast, although both biomarkers were independently correlated to outcome, NSE had limited additional predictive capabilities in the presence of S100B in multivariate models, due to covariance between the two biomarkers (correlation coefficient 0.673 for AUC 48 hours). Moreover, NSE was to a greater extent correlated to multitrauma the first 48 hours following injury, whereas the effect of extracerebral trauma on S100B levels appears limited to the first 12 hours.

**Conclusions:**

While both biomarkers are independently correlated to long-term functional outcome, S100B is found a more accurate outcome predictor and possibly a more clinically useful biomarker than NSE for TBI patients.

## Background

Worldwide, traumatic brain injury (TBI) is a major cause of death and disability with an estimate of 10 million affected annually, among whom many survive but with lifelong disabilities [1]. Due to sociodemographic changes, the burden of TBI is changing more from the young to the already frail elderly, with increasing costs for society [2, 3]. Patients suffering from severe TBI are, often following neurosurgical intervention, usually sedated and treated in neuro-intensive care units (NICU) where they are monitored and treated in order to optimize intracranial conditions and facilitate recovery. Parameters such as intracranial pressure (ICP) [4], metabolism (using microdialysis monitoring) [5], and brain oxygen saturation [6] are monitored and changes may prompt responses in treatment strategies. Unfortunately, many TBI patients still suffer from secondary insults that may result in persistent secondary injuries [7, 8]. Thus, better monitoring, outcome prediction, and injury stratification is necessary in order to optimize resource allocation, to guide treatment, and to prevent further deterioration.

In order to facilitate this, serum biomarkers have been introduced in the field of TBI, where the proteins S100B and neuron-specific enolase (NSE) are the most studied [9]. S100B is a calcium-binding protein present primarily in the cytoplasm of mature perivascular astrocytes [10]. Increased serum levels of S100B in serum have been correlated to pathology on computed tomography (CT) scans in mild TBI [11], to unfavorable long-term functional outcome following moderate-to-severe TBI [12–14] and may indicate a development of secondary injuries in TBI patients [15, 16]. However, S100B is also present in other nonneuronal tissue, primarily in melanocytes, adipose tissue, cartilage, bone, liver, and myocytes [17]. NSE is an enzyme involved in glycolysis in both neuronal cells and erythrocytes, and elevated serum levels have been shown to correlate to unfavorable outcome and clinical complications in the NICU [18–21]. Biomarkers have the advantage of being more “global” monitoring markers of brain injury than microdialysis and oxygen saturation, which only detects changes in a limited cerebral region. However, neither S100B or NSE are 100 % brain specific, and extracranial sources will contribute to total serum levels if patients suffer from severe multitrauma or, in the case of NSE, hemolysis [22–27]. In aggregate, while both S100B and NSE shows promising results as predictive markers of outcome and brain injury severity, they appear to be nonspecific to TBI and the effect of this on their interpretation needs to more thoroughly investigated.

The timing of S100B sampling in relation to that of the trauma plays a crucial role when it is used to predict long-term outcome, and several current studies suggest that samples acquired hours or even days after trauma are better for outcome prediction than samples acquired at admission [12, 18, 28, 29]. In contrast, less is known about how the predictive power of NSE changes over time [18]. The serum half-life for S100B, without cerebral contribution, is considered to be around 25 minutes while that of NSE is 30 hours [24, 30], which theoretically should influence outcome predictive capabilities and optimal timing of sampling.

At our institution, we have sampled both S100B and NSE routinely since 2005 resulting in an extensive database comprising, to our knowledge, the largest clinical set of NSE and S100B in TBI patients to date.

## Methods

### Aims

The primary aim of this study was to assess how serum NSE, independently and together with S100B, correlated to long-term functional outcome. As a secondary aim, we investigated how multitrauma and other factors influenced the biomarker levels over time.

### Study design

This is a database study of S100B and NSE levels collected prospectively from patients admitted to the NICU at Karolinska University Hospital from January 1, 2005 to December 31, 2013. The current study was approved by the Regional Ethical Review Board in Stockholm County (#2014/791-31/1). Using the TBI database registered by the Department of Clinical Neuroscience, Section for Neurosurgery, there were 1128 patients between 2005 and 2013 with an International Classification of Disease (ICD) 10 code S06.XX (traumatic intracranial injury). Inclusion criteria were; age ≥18 years, at least three measurements of S100B and NSE, where the first sample had to be obtained within 48 hours and three samples within 72 hours after trauma. Moreover, the admission CT scan had to be available and the long-term functional outcome had to be evaluated ≥3 months after the trauma. Parts of this patient material have been used in three previous publications by our group, then focusing on S100B and neurofilament light [12, 15, 31].

### Treatment

At our NICU, we adhere to guidelines similar to that of the Brain Trauma Foundation [32, 33]. If mass lesions are present, they are evacuated if deemed appropriate by the attending neurosurgeon. To measure intracranial pressure (ICP), ventricular catheters were predominantly used, even if other pressure devices were sometimes utilized (Codman Neuro, DePuy Synthes Companies of Johnson & Johnson, New Brunswick, NJ, USA or Rehau AG & Co, Bern, Germany). The ICP was targeted below the threshold of 20 mmHg. The head of the patients was elevated at a 30-degree angle with the measuring device set at the temple. In case of intracranial hypertension or autonomic dysfunction, cerebral perfusion pressure (CPP) was used to guide treatment, targeted at 50–70 mmHg calculated by using mean arterial pressure (MAP) – ICP. CPP control was obtained by using vasopressors or intravascular infusions. Unconscious patients were intubated, mechanically ventilated, and anesthetized with morphine, propofol, or midazolam. For patients with refractory high ICP, barbiturate coma (monitored and limited by burst-suppression on electroencephalogram) or hemicraniectomy was performed. Patients with traumatic subarachnoid hemorrhage (trSAH) were monitored with transcranial doppler and if signs of vasospasms were detected, treated with intravascular infusion of the calcium antagonist nimodipine [34]. Body temperature was targeted at 36–37 °C, regulated predominantly with paracetamol, and occasionally parecoxib or Thermowrap® treatment (MTRE Advanced Technologies Ltd., Yavne, Israel).

### Clinical parameters

Scene-of-accident hypoxemia was defined as an oxygen saturation <90 % and hypotension as a systolic blood pressure <90 mmHg. Age and gender were noted. Multitrauma was defined according to Advanced Trauma and Life Support (ATLS) guidelines, with a trauma to any other organ apart from head or cervical injuries [35]. In contrast to the Abbreviated Injury Score (AIS) [36], it provides a cruder measurement of extracranial trauma. However, as AIS is a product of events during the hospital stay, we believe this to be a more accurate definition in the emergency setting. Admission glucose and hemoglobin were recorded. If fluids had been provided in the prehospital setting, hemoglobin levels at the scene of accident were used, if available. Glasgow Coma Scale (GCS) [37] was assessed at hospital admission and was used as a continuous variable as previously described [38, 39]. Pupil responsiveness was assessed as either both responsive, unilateral unresponsive or bilateral unresponsive. The maximum AIS score for the head injury was noted for each patient after discharge, assessed by nurses with special training in AIS grading [36].

### Neuroradiology

The admission CT scan was assessed according to the Marshall Classification [40], as well as the Rotterdam and Stockholm CT score [41, 42], by a person blinded to other clinical data. The Marshall classification is more focused on the type of injury while the Rotterdam and Stockholm CT scores focus on parameters correlated to outcome. The Stockholm CT score has a subcomponent of assessing the degree of subarachnoid hemorrhage (Stockholm SAH), which was also used in isolation in the analyses. The time from reported trauma to CT examination was noted.

### S100B and NSE analyses

As per clinical protocol since 2005, all TBI patients in our NICU have S100B and NSE sampled at admission and twice daily (06:00 and 18:00). From 2005 up until September 2008, the serum samples of S100B were analyzed at the Department of Clinical Chemistry, Karolinska University Hospital (fully blinded from any patient characteristic), using a quantitative automated immunoassay (LIAISON, DiaSorin, Saluggia, Italy). After that, the department changed method to an automated electrochemoluminescence assay (Modular E170, Elecsys, Roche Diagnostics, Basel, Switzerland) throughout the study period. S100B has been shown not to be significantly influenced by sample hemolysis [25].

NSE was analyzed throughout the whole period at the Department of Clinical Chemistry, Karolinska University Hospital, using a quantitative automated immunoassay (LIAISON, DiaSorin, Saluggia, Italy). Samples were not analyzed if the amount of hemoglobin exceeded 0.5 g/L which was assessed visually using a hemolysis scale. If the laboratory personnel determined that the amount of hemoglobin exceeded 0.5 g/L, the sample was discarded. The NSE samples were acquired simultaneously as S100B.

The samples were sent to the Department of Clinical Chemistry for immediate analysis. The admission sample was usually from a venous source while the subsequent samples were from arterial lines. The first three samples and the area under curve (AUC), calculated using these samples during the first 48 hours, were used. The lowest levels of detection (LLOD) are 0.02 μg/L for S100B and 0.04 μg/L for NSE on the LIAISON assay, and <0.005 μg/L for S100B on the Elecsys device. However, the lowest level of quantification (LLOQ, also known as functional sensitivity) has been shown to be 0.02 μg/L and 0.04 μg/L for S100B and NSE on the LIAISON, respectively, as well as <0.02 μg/L on the Elecsys assay, while lower concentrations sometimes also yield acceptable coefficients of variation [43–45]. Thus, all detection and quantification limits were lower than what were detected in our patient material, and we do not believe that the quantification range of the assays significantly altered our findings. The reference levels for healthy controls are generally considered to be <0.1 μg/L for S100B [11] and <13 μg/L for NSE [46], respectively.

### Long-term functional outcome

At our institution, the five-step Glasgow Outcome Score (GOS) [47] is assessed at discharge, after 3–6 months as the patient visits the operating physician in the clinic or at the rehabilitation facility and after 12 months through a questionnaire regarding quality of life. GOS1 = dead, GOS2 = vegetative state, GOS3 = severe, dependent state, GOS4 = moderately recovered, independent state and GOS5 = good recovery. The latest available GOS was used and surviving patients with GOS evaluation <3 months after trauma were excluded.

### Statistical analysis

The collected data are presented as median and interquartile range (IQR) for continuous data and grouped for categorical data. Several parameters were not normally distributed, including the biomarkers, and were logged to approach to normal distribution. In univariate logistic regression toward outcome (“rms” package in R), the parameters were analyzed individually versus either GOS1–5 (proportional odds analysis), GOS1–3 (unfavorable) versus GOS4–5 (favorable), or GOS1 (dead) versus GOS2–5 (alive). A step-up multivariate analysis was used to determine if different parameters added significantly explained the variance to the different models. We included the same parameters that are used in the International Mission for Prognosis and Analysis of Clinical Trials in TBI (IMPACT) calculator to determine long-term functional outcome after TBI [48]. Nagelkerke’s pseudo-R^2^ was used to determine model accuracy of binomial and proportional odds analyses models and adjusted R^2^ that of linear correlations, where appropriate. A sliding window assessing a proportional odds analysis of S100B and NSE toward GOS with bootstrapped confidence intervals used to explore prediction accuracy over time in relation to the trauma. The same approach was used to assess how multitrauma influenced S100B and NSE levels over time. The R package “ggplot2” was used to illustrate the data [49]. Linear models were used to determine what factors that contributed both in univariate and in multivariate analyses to the levels of NSE and S100B, and the resulting adjusted R^2^ was provided. The statistical program R was used in the analyses (RStudio version 0.99.486 using version 3.2.2 of R, Boston, MA, USA) [50]. A *p* value of <0.05 was considered significant.

### Missing data

Univariate regression models toward outcome are shown for un-imputed data. Multivariate prediction models were performed using the Multiple Imputation (MI) (MICE package, R), as advocated in the statistical literature [51] and suggested by the IMPACT TBI study group [52]. MI commonly uses seven imputed sets of data, where the imputed data comes from a regression and each imputed data differs - drawn from a distribution. The purpose of this is to retain the uncertainty caused by imputation in the analyses. This method has been shown to handle up to 50 % imputed data with limited introduction of bias. Unfortunately, AIS grading started in January 2006 at our institution so this information was not available for patients in 2005 (*n* = 72) and was among the data that was more highly imputed, and will due to the time component, to some extent, violate the missing at random assumption.

## Results

### Patient characteristics

Out of 1128 patients, 417 patients fulfilled the inclusion criteria. A majority of the excluded patients did not have enough samples (*n* = 593), while some were <18 years old (*n* = 47), had too early a GOS assessment (*n* = 35), had missing time of trauma (n = 31), or an admission CT scan from another primary hospital that could not be obtained (*n* = 5). Among the 593 patients with too few samples, 24 patients were excluded due to early death. Patient demographics are illustrated in Table 1. Our inclusion criteria excluding patients with short ICU stay and predominantly higher GCS patients rendered almost all eligible patients to be classified as Head AIS ≥3, or a “serious” TBI, which coincided with that a majority also were unconscious at admission to the hospital (GCS3–8, 66 %). “Diffuse injury”, according to the Marshall CT classification, was present in 35 %, while 65 % had injuries >25 mL (“focal injury”, grade VI). Almost 20 % of the patients had hemolysis in their NSE samples the first 72 hours. Outcome was assessed at approximately 1 year in a majority of cases (median days from trauma = 368), and was almost equally distributed between favorable (GOS4–5) (*n* = 214, 51 %) and unfavorable (GOS1–3) (*n* = 203, 49 %). The mortality rate of the included patients was 20 %.

**Table 1:** Patient demographics

Parameter	Category	Data	Missing, *n* (%)
Age, years	Median (IQR)	52 (34–62)	
Gender	Male/female, *n* (%)	332/85 (80/20)	
Scene of accident			
Multitrauma	*n* (%)	131 (31)	2 (0.5)
Hypoxemia (oxygen saturation <90 %)	*n* (%)	47 (11)	115 (28)
Hypotension (systolic blood pressure <90 mmHg)	*n* (%)	11 (3)	118 (28)
Admission			
Glasgow Coma Score (GCS)	GCS 3–8, *n* (%)	274 (66)	
	GCS 9–13, *n* (%)	105 (25)	
	GCS 14–15, *n* (%)	38 (9)	
Pupil unresponsiveness,	Out of total, *n* (%)	93 (22)	13 (3)
	Unilateral unresponsiveness, *n* (%)	53 (13)	
	Bilateral unresponsiveness, *n* (%)	40 (10)	
Hemoglobin (g/L)	Median (IQR)	136 (121–147)	19 (5)
Glucose (mmol/L)	Median (IQR)	8.0 (7.0–9.8)	99 (24)
Time from trauma to sampling (hh:mm)	Median (IQR)	01:05 (00:45–03:39)	
Head Abbreviated Injury Score (AIS)	2, *n* (%)	1 (0.2)	77 (18)
	3, *n* (%)	40 (10)	
	4, *n* (%)	119 (29)	
	5, *n* (%)	177 (42)	
	6, *n* (%)	3 (1)	
Radiology			
Marshall CT Classification	I (Diffuse injury), *n* (%)	1 (0.2)	
	II (Diffuse injury), *n* (%)	110 (26)	
	III (Diffuse injury), *n* (%)	34 (8)	
	IV (Diffuse injury), *n* (%)	1 (0.2)	
	VI (Focal injury), n (%)	271 (65)	
Rotterdam CT Score	1, *n* (%)	10 (2)	
	2, *n* (%)	39 (9)	
	3, *n* (%)	155 (37)	
	4, *n* (%)	116 (28)	
	5, *n* (%)	80 (19)	
	6, *n* (%)	17 (4)	
Stockholm Score	Median (IQR)	2.5 (2.0–3.5)	
Time from trauma to examination (hh:mm)	Median (IQR)	01:32 (01:09–02:23)	
Biomarkers			
Time from trauma to admission sample (hh:mm)	Median (IQR)	07:14 (02:40–13:27)	
S100B (μg/L), admission	Median (IQR)	0.57 (0.26–1.4)	
Neuron-specific enolase (NSE) (μg/L), admission	Median (IQR)	21 (15–31)	
Time from trauma to second sample (hh:mm)	Median (IQR)	17:30 (10:51–26:39)	
S100B (μg/L), second sample	Median (IQR)	0.38 (0.20–0.78)	
NSE (μg/L), second admission	Median (IQR)	19 (14–26)	
Time from trauma to third sample (hh:mm)	Median (IQR)	30:29 (22:30–42:09)	
S100B (μg/L), third sample	Median (IQR)	0.32 (0.15–0.74)	
NSE (μg/L), third admission	Median (IQR)	17 (12–24)	
Patients with NSE hemolysis the first 72 hours	*n* (%)	75 (18)	
Outcome			
Time to outcome assessment in surviving patients (days)	Median (IQR)	368 (339–397)	
Glasgow Outcome Score (GOS)	GOS1, *n* (%)	85 (20)	
	GOS2, *n* (%)	2 (0.5)	
	GOS3, *n* (%)	116 (28)	
	GOS4, *n* (%)	126 (30)	
	GOS5, *n* (%)	88 (21)	
	GOS1–3 (Unfavorable), *n* (%)	203 (49)	
	GOS4–5 (Favorable), *n* (%)	214 (51)	

Demographics for the included 417 patients categorized in parameters acquired at scene of accident, admission, neuroradiology and biomarker data as well as long-term outcome. Number of missing samples is listed in the right column

### Univariate analyses toward long-term outcome

**Fig. 1:**
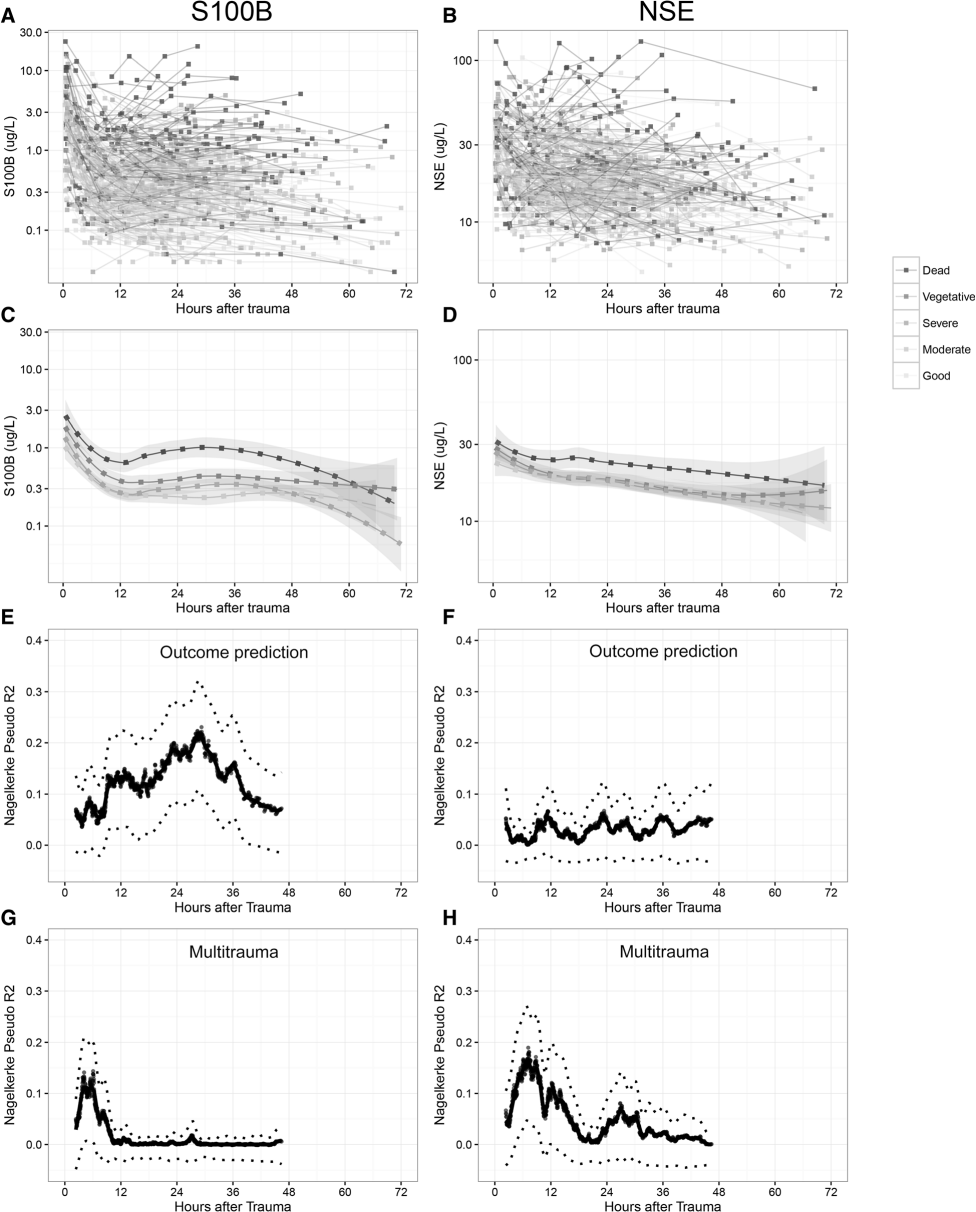
**a** and **b** illustrate every patient as an individual line with the biomarker S100B (**a**) and neuron-specific enolase (NSE) (**b**) on the y-axis and time after trauma on the x-axis (hours). Colors are corresponding to outcome with darker color indicating a worse outcome, which becomes more favorable as it gets lighter. **c** and **d** are averages of the different GOS groups. As is shown by (**a** and **b**), there is limited data after 48 hours so it should be interpreted with caution. **e** and **f** are line plots indicating when to sample a biomarker after trauma to achieve maximum outcome prediction to long-term GOS1–5. The x-axis shows when in time since the trauma the sample of S100B (**e**) and NSE (**f**) was acquired (hours). The y-axis represents the Nagelkerke’s pseudo-R^2^ of a prediction model (proportional odds) toward GOS1–5, using either logged S100B (**e**) or NSE (**f**). The pseudo-R^2^ is calculated in each point using a sliding window incorporating 200 data points in chronological order. If a patient is represented more than once the sample is averaged, thus retaining independent points. The graph stops at approximately 48 hours as the later data points will be included in that final measurement. The line represents a locally weighted scatterplot smoothing (LOWESS), which is a nonlinear regression of the data points in the plots, a bootstrap confidence interval using two standard deviations is provided. Finally, in (**g** and **h**), which use the same method as in (**e** and **f**), but here the explained variance (y-axis) is how well the presence of extracranial multitrauma explains the levels of S100B (**g**) and NSE (**h**)

**Fig. 2:**
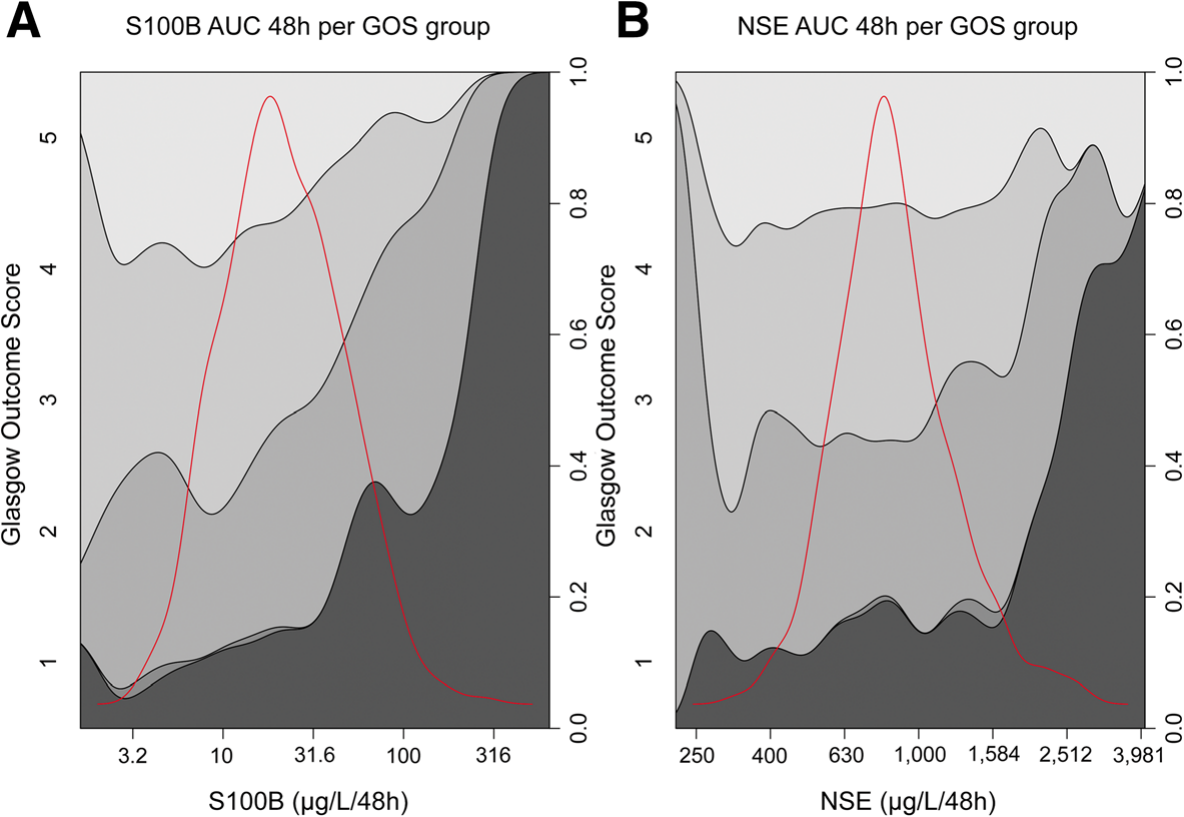
Conditional density plots of S100B (**a**) and neuron-specific enolase (NSE) (**b**) area under curve (AUC) 48 h per Glasgow Outcome Score (GOS) group. Log biomarker AUC data is provided on the x-axis (μg/L/48 hours). The numbers on the left y-axis represent GOS while outcome proportions, summing to one is on the right y-axis. An overlay indicates the distribution of S100B and NSE samples

Known outcome predictors of TBI such as age, pupil responsiveness, and GCS were all significant in univariate analysis and had an expected high pseudo-R^2^ (Table 2). S100B AUC 48 h exhibited a pseudo-R^2^ of 0.132, on par with Stockholm CT score and surpassed only by age (0.151). S100B showed better discrimination between all the different dichotomizations of outcome, whereas NSE is best at differentiating mortality (GOS1 vs. 2–5). The values of S100B and NSE over time for individual patients are shown in Fig. 1. In general, high or increasing levels are more correlated to a more unfavorable outcome, something that is better visualized for S100B (Fig. 1a) than for NSE (Fig. 1b). Similar to what was seen in Table 2, when the biomarker levels for the specific outcome groups were aggregated, S100B exhibits better discrimination between different levels of GOS, especially 24–36 hours after trauma (Fig. 1c), while NSE was only discriminates GOS1 as compared to GOS3–5 (Fig. 1d). GOS2 was excluded in Fig. 1c-d since only two patients were assessed as vegetative at long-term follow-up. S100B AUC 48 h (Fig. 2a) and NSE AUC 48 h (Fig. 2b) highlights the biomarker levels in different GOS groups, using conditional density plots (Fig. 2).

**Table 2:** Univariate outcome prediction

	GOS 1–5 (proportional odds analysis)	
	*p* value	Nagelkerke’s pseudo-R^2^ (coefficient)
Gender (female)	0.760	0.000 (-)
Age	**<0.001**	**0.151 (-, higher age = lower GOS)**
Pupil unresponsiveness	**<0.001**	**0.074 (-, if present = lower GOS)**
Glasgow Coma Score (GCS)	**<0.001**	**0.070 (+, higher GCS = higher GOS)**
Multitrauma	0.867	0.000 (+)
Hypoxemia	**0.040**	**0.015 (-)**
Hypotension	0.494	0.002 (-)
Glucose	**0.010**	**0.022 (-)**
Hemoglobin	**<0.001**	**0.044 (+)**
Head Abbreviated Injury Score (AIS)	**0.002**	**0.029 (-)**
Marshall	0.190	0.004 (-)
Rotterdam	**<0.001**	**0.048 (-)**
Stockholm	**<0.001**	**0.132 (-)**
Stockholm subarachnoid hemorrhage (SAH)	**<0.001**	**0.079 (-)**
S100B admission (log)	**<0.001**	**0.051 (-)**
Neuron-specific enolase (NSE) admission (log)	**0.025**	**0.013 (-)**
S100B area under the curve (AUC) 48 h (log)	**<0.001**	**0.132 (-)**
NSE AUC 48 h (log)	**0.001**	**0.038 (-)**
	GOS 1–3 vs 4–5 (bivariate regression analysis)	
S100B admission (log)	**<0.001**	**0.061 (-)**
NSE admission (log)	0.096	0.009 (-)
S100B AUC 48 h (log)	**<0.001**	**0.127 (-)**
NSE AUC 48 h (log)	**0.002**	**0.032 (-)**
	GOS 1 vs 2–5 (bivariate regression analysis)	
S100B admission (log)	**0.001**	**0.054 (-)**
NSE admission (log)	**0.008**	**0.027 (-)**
S100B AUC 48 h (log)	**<0.001**	**0.179 (-)**
NSE AUC 48 h (log)	**<0.001**	**0.077 (-)**

Table illustrating different un-imputed parameters versus different outcome dichotomizations. Nagelkerke’s pseudo-R^2^ and regression coefficients are shown to facilitate interpretation. A negative coefficient means that a higher level of the parameter correlated to a lower Glasgow Outcome Score (GOS) (e.g., age) and vice versa (e.g., GCS). Bold indicates significance (*p* <0.05)

### Optimal time point for outcome prediction

A sliding window using a proportional odds analysis toward GOS1–5 indicated that the level of S100B’s relation to outcome increased substantially after 12 hours (as previously described [12]) and reached a pseudo-R^2^ of >0.25 at approximately 30 hours (Fig. 1e), coinciding with the mean peak time of the extended release of S100B in TBI patients at 27 hours (accepted manuscript). In contrast, an optimal time point for sampling NSE in relation to outcome could not be identified and remained low, <0.1 pseudo-R^2^ (Fig. 1f), at all time points. Additionally, the effect of multitrauma on biomarker levels over time was explored in the same manner with a sliding window, revealing that the effect of multitrauma is limited to the first 12 hours for S100B, but is more pronounced and extended for NSE, (Fig. 1g and h) clearly indicating a risk of late confounding using this biomarker.

### Multivariate prediction models toward long-term functional outcome

A Core model was created using the parameters used in the IMPACT calculator (using the Rotterdam CT score instead of individual CT parameters) [48] (Table 3). This exhibited a pseudo-R^2^ of 0.298 in prediction of GOS1–5. As the Stockholm CT score constituted a higher pseudo-R^2^ in univariate models, it replaced the Rotterdam CT score in our Core model, which thus yielded a total pseudo-R^2^ of 0.316. AUC levels of the biomarkers were used. If S100B AUC was added to the Core model, a pseudo-R^2^ of 0.379 was reached, significantly better than the Core model (*p* <0.001). A similar significant increase was seen if NSE AUC was added per se, albeit less than S100B (0.379 vs 0.344 respectively). A glial:neuronal ratio between S100B:NSE (S100B AUC/NSE AUC) did not increase the explained variance as compared to if S100B was used alone (pseudo-R^2^ 0.365). If a prediction model consisted of Core parameters + S100B, NSE did not enhance outcome prediction (*p* = 0.934). Age, pupil responsiveness, GCS, and Stockholm CT score remained independently correlated to outcome in all models while glucose levels, hemoglobin levels, and hypoxemia contributed independently variably in models. In aggregate, the Core model with the addition of S100B AUC was the best predictive model.

**Table 3:** Multivariate outcome prediction models

	Parameters included	Explained variance (pseudo-R^2^)
IMPACT	**Age** + **pupils** + **GCS** + hypoxemia + hypotension + Hb + **glucose** + Rotterdam CT score	0.298
Core	**Age** + **pupils** + **GCS** + **hypoxemia** + hypotension + Hb + **glucose** + **Stockholm CT score**	0.316
Core + S100B	**Age** + **pupils** + **GCS** + hypoxemia + hypotension + Hb + glucose + **Stockholm CT score** + **S100B AUC 48 h**	0.379 (*p* <0.001 vs Core)
Core + neuron-specific enolase (NSE)	**Age** + **pupils** + **GCS** + hypoxemia + hypotension + **Hb** + glucose + **Stockholm CT score** + **NSE AUC 48 h**	0.344 (*p* <0.001 vs Core)
Core + (S100B/NSE)	**Age** + **pupils** + **GCS** + hypoxemia + hypotension + Hb + **glucose** + **Stockholm CT score** + (**S100B AUC 48 h/NSE AUC 48 h**)	0.365 (*p* <0.001 vs Core)
Core + S100B + NSE	**Age** + **pupils** + **GCS** + hypoxemia + hypotension + Hb + glucose + **Stockholm CT score** + **S100B AUC 48 h** + NSE AUC 48 h	0.379 (*p* = 0.934 vs Core + S100B)

Table showing the different multivariate models to predict GOS 1–5. Bold indicates which parameters that were independently correlated to outcome in that specific model. A “Core” model was created, similar to the IMPACT calculator but with Stockholm CT score instead of Rotterdam CT score

### Correlation between S100B and NSE levels

Admission levels of logged S100B and NSE were significantly correlated with a correlation coefficient of 0.673 (*p* <0.001, R^2^ 0.45). A weaker correlation was seen for logged AUC levels the first 48 hours after trauma, yielding a correlation coefficient of 0.613 (*p* <0.001, R^2^ 0.38).

### Parameters correlated to S100B and NSE levels

A univariate analysis revealed that several parameters were significantly correlated to the levels of NSE and S100B, as illustrated by Table 4. For multivariate analysis, the Stockholm SAH score was used instead of Stockholm CT score, as it provided a higher R^2^. Several parameters were independently correlated to NSE levels, while only pupil unresponsiveness, hypoxemia, and higher Stockholm SAH score independently predicted S100B levels. Admission levels for NSE and S100B exhibited a different profile, with no intracranial CT parameter being significantly correlated to NSE and multitrauma being positively correlated to S100B (data not shown). Using all parameters, including intracranial variables, the best model explained 0.195 adjusted-R^2^ of NSE and 0.161 of S100B, thus concluding that much of the variance remains unaccounted.

**Table 4:** Parameters correlated to S100B and NSE levels

	NSE AUC 48 h		S100B AUC 48 h	
	*p* value	R^2^ (coefficient)	*p* value	R^2^ (coefficient)
Gender	0.937	0.000 (-)	0.862	0.000 (+)
Age	**<0.001**	^*^ **0.028 (-)**	0.055	0.009 (+)
Pupil unresponsiveness	**<0.001**	^*^ **0.030 (+)**	**<0.001**	^*^ **0.032 (+)**
Glasgow Coma Score (GCS) admission	**0.001**	**0.024 (-)**	**<0.001**	**0.028 (-)**
Multitrauma	**<0.001**	^*^ **0.032 (+)**	0.202	0.004 (+)
Hypoxemia scene of accident (SoA)	**0.015**	**0.014 (+)**	**<0.001**	^*^ **0.038 (+)**
Hypotension SoA	0.237	0.003 (+)	0.430	0.001 (+)
Glucose admission	**0.022**	**0.012 (+)**	**0.005**	**0.018 (+)**
Hemoglobin admission	0.072	0.008 (+)	0.258	0.003 (-)
Head Abbreviated Injury Score (AIS)	0.123	0.006 (+)	**0.033**	**0.011 (+)**
Marshall CT classification	0.931	0.000 (+)	0.207	0.004 (+)
Rotterdam CT score	**0.006**	**0.018 (+)**	**<0.001**	**0.045 (+)**
Stockholm CT score	**0.001**	**0.029 (+)**	**<0.001**	**0.060 (+)**
Stockholm subarachnoid hemorrhage (SAH) score	**<0.001**	^*^ **0.074 (+)**	**<0.001**	^*^ **0.098 (+)**
Model explained (adjusted R^2^)		0.195		0.161

Table illustrating which parameters that were correlated to S100B and neuron-specific enolase (NSE) levels using linear univariate and multivariate models. Bold indicates significance (*p* <0.05). The asterisk (^*^) highlights which parameters were independently correlated to the levels of each biomarker. Adjusted-R^2^ and coefficient are shown to facilitate comparison and interpretation

## Discussion

To our knowledge, this is the largest retrospective TBI outcome study including S100B and NSE samples to date. Moreover, by combining the two proteins in the analyses, it provides a unique opportunity to elucidate the properties and clinical utility of these biomarkers. Doing this we found that both NSE and S100B levels, per se, independently correlate to long-term functional outcome in univariate and multivariate models, albeit S100B providing significantly more accuracy. While patients with favorable outcome have low and rapidly decreasing levels, S100B and NSE levels in patients with unfavorable outcome remain elevated during a longer period of time, presumably indicating an ongoing cerebral injury. However, in combination NSE did not provide any additional independent information toward long-term outcome over S100B. The cause of this is that despite their neuronal versus glial origin, a high covariance between the two biomarkers is seen. Additionally, NSE only discriminates mortality, while S100B provides predictive ability at all levels of GOS. While S100B increased its predictive capabilities if sampled 12–30 h after trauma, NSE’s predictive capabilities remained low at all time points, possibly due to a greater influence by multitrauma or other non-brain contributors to total serum levels such as hemolysis of erythrocytes ex vivo. In aggregate, our study suggests a greater clinical utility of S100B over NSE.

We chose to include all TBI patients that were deemed to be in need of neuro-intensive care at admission to the hospital, and not necessarily patients that were unconscious (GCS 3–8), which are usually grouped together when stratifying TBI patients, and is something that could be considered a limitation. However, there is a strong opinion in the TBI community today that using a symptom to include TBI patients in studies is inadequate. A major conclusion of the IMPACT group was that GCS inclusion criterion could be a contributor to the many negative clinical trials in the field [53]. This is exemplified in our study, as several of our moderate-to-mild patients (GCS 9–15) at admission presented “serious”, “severe” or even “critical” injuries according to their Head AIS scores. Moreover, GCS is also hazardous due its subjective nature [54], as well as its influence from drugs, ethanol, and sedative agents [55, 56]. In aggregate, we deem that the best inclusion criteria is the one that is currently used, i.e., that patients who the attending neurosurgeon deem will be in need of neuro-intensive care and intracranial monitoring for intracranial injuries, and that this represents a clinically valid NICU TBI patient cohort.

### Outcome prediction using S100B and NSE

That our outcome models found NSE levels correlated to outcome in univariate, as well as independently in multivariate analyses, is congruent with earlier studies [57, 58]. However, in contrast to S100B, NSE does not appear to discriminate between favorable and unfavorable outcome, but only mortality versus survival, something that is supported by similar findings in a recent meta-analysis of NSE [21] and by Vos and co-workers [39]. When compared to NSE, S100B has a higher overall predictive power, in accordance with other groups analyzing and comparing both biomarkers [59–61], where the predictive capability of NSE was found limited in the presence of S100B in multivariate outcome models [39, 62]. An explanation is, again, the notable covariance between serum S100B and NSE, which has been shown to be 0.50–0.78 (correlation coefficient) in previous studies [39, 58, 63, 64], similar to ours of 0.67. However, in our study this correlation decreases over time, highlighting the need for more granular temporal considerations when assessing biomarkers [65]. That NSE holds similar information as S100B, suggests that they are part of a similar pathophysiological process despite their separate cellular origins. This contrasts with, for example, neurofilament light (NF-L), which has seen to have a much lower covariance with S100B [31]. In aggregate, while NSE is an independent outcome predictor in TBI, it does not add any additional pseudo-R^2^ in the presence of S100B, which is a better predictor overall.

### Optimal sampling time to assess outcome

Timing of biomarker sampling in studies is not standardized and much of the differences in findings of studies may relate to dissimilar sampling times. In this study we attempt to focus on some little-studied temporal aspects of these biomarkers. Our results suggest that a more granular focus on temporal changes may be needed in biomarker research in general, as important aspects of characterization may be otherwise lost [65]. The optimal timing of NSE sampling for outcome prediction has acquired insufficient attention earlier. Some studies have only used one sample at admission [66, 67], while others have sampled NSE more frequently and noticed that peak levels are better than admission levels for outcome prediction [18, 62]. We have previously shown that the outcome predictive power of S100B increased substantially after about 12 hours [12]. Again, in this larger cohort, we noticed a similar pattern with an explained variance toward outcome of about 0.25–0.30 some 30 hours after trauma. However, it must be noted that this study overlaps patients (2005–2009) with the current study. In contrast, NSE, exhibits a rather flat and low predictive power over time, with a pseudo-R^2^ < 0.10. Presumably, due to the longer half-life of NSE, NSE will remain elevated for a longer period of time compared to S100B (30 hours vs 25 minutes) [24, 30]. This, and the fact that hemolysis could presumably affect NSE over a prolonged period of time, are potential confounders possibly affecting its predictive capabilities. In summary, both biomarkers are influenced by multitrauma early after injury, however 10–12 hours after trauma, S100B’s predictive capability increases while NSE’s outcome prediction remains relatively low days after trauma.

### Influence of multitrauma on S100B and NSE

In this study, we explored the impact of multitrauma on NSE and S100B levels over time. We used a sliding window methodology clearly suggesting that the effect of multitrauma on S100B is limited to the first 12 hours, whereas in the case of NSE, the correlation between NSE and multitrauma remains past 24 hours. Other studies have found a correlation between NSE and extracranial injury, questioning its validity as a biomarker of cerebral injury [68, 69]. S100B has also been criticized for being released from extracranial sources [22, 23, 70–72]. However, our study indicates that the extracranial contribution is probably more problematic for NSE than for S100B. The washout effect of S100B from extracranial trauma after TBI has been shown to be relatively fast [23, 70], while in theory, several hematomas containing slowly degrading erythrocytes will be contributing to the total NSE levels for days or even weeks, especially given the longer half-life. As can be seen in Fig. 1h, the multitrauma contribution of NSE is increased past 24 hours, which almost coincides with the described serum half-life of the protein [24]. Attempts to adjust for hemolysis in NSE samples have been made, which have shown to yield more accurate results [73, 74], something that unfortunately was not possible in our retrospective approach. In future studies, it would be of interest to isolate the kind of extracranial injury that results in the greatest release of NSE, something that we recently have done with S100B in bicycle injuries [27]. Moreover, in addition to intra- and extracranial injuries, we found NSE to also be significantly correlated with age (negative correlation), an observation that may require further investigation, but we believe that it is due to the fact that a majority of the multitrauma patients were younger. In aggregate, NSE appears more confounded by extracranial trauma than S100B.

### S100B and NSE versus more novel markers of brain injury

More specific protein biomarkers of brain tissue fate in serum are currently being explored, including, among others, glial fibrillary acid protein (GFAP) [75, 76], ubiquitin C-terminal hydrolase L1 (UCH-L1) [77, 78] and neurofilament light (NF-L) [31]. While GFAP have been shown to be less influenced by extracranial injury than S100B in mild TBI [75], both GFAP [79] and UCH-L1 [80, 81] serum levels have been shown to be elevated in trauma patients without head injury. As yet, it is difficult to say if these novel markers are better in predicting outcome and assessing injury severity, as compared to S100B and NSE. Vos and co-workers analyzed S100B, NSE, and GFAP in more severe TBI patients and noted that all exhibited similar capabilities, albeit S100B being a somewhat better predictor of mortality [39]. Similar results have been shown by Pelinka et al. [76], who albeit found GFAP to be a somewhat more accurate outcome predictor than S100B. In mild TBI, S100B has been shown to be a more reliable marker than UCH-L1 as a predictor of injury severity [82]. More importantly, GFAP and UCH-L1 have not been as extensively studied as S100B and NSE in more severe TBI cohorts, and a recent study indicates that UCH-L1 and GFAP may not add predictive power to commonly used prognostic models in TBI [83]. Thus, while more brain-specific proteins exist and are being investigated, studies have yet to confidently show that they are better predictors of TBI outcome and severity than S100B and NSE.

In summary, this study suggests S100B to be the more useful of the two biomarkers for outcome prediction in NICU TBI patients. It is important to note that our findings cannot be translated to other cerebral pathologies such as anoxic brain injuries after cardiopulmonary resuscitation (CPR), or stroke, where NSE is utilized [84–86]. In TBI cohorts similar to ours, early samples of both biomarkers should be interpreted with caution as they are influenced by extracranial trauma. However, in the case for S100B the extracranial trauma component appears negligible from 12 hours after injury. While more brain-specific markers of tissue injury exist, to date, none have in our opinion yet added more utility than S100B in TBI patients. We therefore suggest that future novel biomarkers should be compared with S100B, and that biomarker evaluation should include time series analysis elucidating temporal aspects of information content and possible confounders.

### Limitations

There are several limitations to this study that should be noted. The retrospective nature is associated with some inherent weaknesses, even if sampling at the NICU was done prospectively by clinical protocol for all patients. A total of 593 patients were excluded due to a limited amount of protein biomarker samples. The cause is largely due to patients being treated at other ICUs prior to NICU referral. A majority of these patients did not reach the NICU in time to have three 12-hourly, samples acquired within 48 hours of trauma. As previously noted, the mortality was not higher in this group. Although this clearly affects the cohort composition selecting a more severe TBI population with early referrals and more isolated TBI, we believe that this still represents a clinically relevant NICU population in which to study these biomarkers.

There is a potential treatment bias as levels of NSE and S100B were not blinded to caregivers. While we have not changed any local guidelines due to NSE or S100B sampling, secondary peaks of S100B do trigger further diagnostics and a potential change in the treatment regime [15]. That NSE and S100B are used differentially could affect results, but are in our opinion more the result of years of experience of these biomarkers simultaneously where S100B has emerged as the more clinically comprehensive and consistent biomarker. As we have used biomarkers from the first 72 hours after trauma in this study, and consider these levels more correlated to the initial traumatic cerebral injury than to secondary injuries, we believe that treatment bias will not meaningfully influence conclusions.

An additional limitation is that we could not in retrospect adjust for the hemolysis contribution [25], something that would have been possible if we had a hemolysis index [74]. The method of ocular inspection of hemolysis used at the laboratory during this study is inherently blunt and subjective. It is difficult to define if the hemolysis index (hemoglobin concentration) is 0.25, 0.50, or even 1.0 g/L hemoglobin in a sample and thus it is fair to assume that hemolysis probably influenced the samples resulting in higher total levels of NSE in some patients. A better hemolysis adjustment may however lead to an even greater covariance between NSE and S100B.

Finally, the S100B assay was changed during the experimental period. While good correlations between the two methods have been shown [43, 44, 87], others have shown that there is a discrepancy, especially in concentrations above 0.4 μg/L, and that the Elecsys device consistently measured lower levels (i.e., a majority of our samples were lower than 0.4 μg/L) [88]. In the current dataset, comparing S100B levels for patients with unfavorable outcome between the two assays (87 of the 170 patients with the old LIAISON assay vs 116 of the 247 patients with the new Elecsys assay) indicated that the LIAISON yielded a median peak level of 1.2 (interquartile range: 0.6–2.3) μg/L while the Elecsys assay 1.0 (interquartile range: 0.3–2.9) μg/L of S100B (*p* = 0.315, Mann-Whitney *U* test). Thus, we could not replicate the significantly lower levels seen in other studies with the current study material, supporting that the assay change is a minor limitation.

## Conclusions

In this largest cohort of biomarkers NSE and S100B to date, we found that NSE is independently correlated to long-term functional outcome in neuro-intensive care-treated TBI patients, but loses its predictive capabilities in the presence of S100B, which is a more accurate outcome predictor in both univariate and multivariate models. This is due to a strong correlation and thus covariance between the two biomarkers, possibly reflecting a similar pathophysiological process, albeit from different cellular origins. Moreover, NSE is seen related to extracranial trauma up to 48 hours after trauma, whereas this influence is limited to 12 hours for S100B. In summary, S100B appears the more useful biomarker of these two in this population. Additionally, we find it important to focus future biomarker studies on temporal relationships of information content, and toward that of confounders, to better understand and evaluate clinical utility. Prospective studies are necessary to better, and with higher accuracy, correlate putative biomarkers to outcome and injury severity.

## Abbreviations

AIS: Abbreviated Injury Score
AUC: Area under the curve
CPP: Cerebral perfusion pressure
CT: Computed tomography
GFAP: Glial fibrillary acidic protein
GCS: Glasgow Coma Scale
GOS: Glasgow Outcome Score
ICP: Intracranial pressure
NF-L: Neurofilament light
NICU: Neuro-intensive care unit
NSE: Neuron-specific enolase
SAH: Subarachnoid hemorrhage
TBI: Traumatic brain injury
UCH-L1: Ubiquitin C-terminal hydrolase L1
